# Retraction: A Novel Alkaloid from Marine-Derived Actinomycete *Streptomyces xinghaiensis* with Broad-Spectrum Antibacterial and Cytotoxic Activities

**DOI:** 10.1371/journal.pone.0200376

**Published:** 2018-07-03

**Authors:** The PLOS ONE Editors

The *PLOS ONE* Editors retract this publication due to concerns about the chemical structure and spectral data reported in this article.

After publication of the article, concerns were raised regarding the validity of the reported structure of Xinghaiamine A, and about the spectral data used to validate that structure.

*PLOS ONE* staff consulted a Section Editor and asked the authors to provide the raw data underlying the results presented in the article. The authors did not provide the original data, but they requested withdrawal of the article and confirmed that the structure presented is incorrect. The corresponding author apologized for the errors and indicated that spectral images from NMR analyses of different purification batches had been combined in error when deducing the reported structures.

In the light of the concerns about the integrity of the results and the underlying data, the *PLOS ONE* editors retract this publication.

WJ, FZ, XZ, JH and J-WS agreed with the retraction.
